# Oral supplementation with yeast *β*-glucans improves the resolution of *Escherichia coli*-associated inflammatory responses independently of monocyte/macrophage immune training

**DOI:** 10.3389/fimmu.2022.1086413

**Published:** 2022-12-20

**Authors:** Sarah Walachowski, Koen Breyne, Thomas Secher, Céline Cougoule, Laurence Guzylack-Piriou, Evelyne Meyer, Gilles Foucras, Guillaume Tabouret

**Affiliations:** ^1^ Interactions Hôtes-Agents Pathogènes (IHAP), Université de Toulouse, ENVT, Institut National de la Recherche Agronomique et Environnement (INRAE), Toulouse, France; ^2^ Molecular Neurogenetics Unit, Neurology and Radiology Department, Massachusetts General Hospital - Harvard Medical School, Charlestown, MA, United States; ^3^ INSERM, Centre d’Etude des Pathologies Respiratoires, Tours, France; ^4^ Faculté de Médecine Université de Tours, Tours, France; ^5^ Institut de Pharmacologie et de Biologie Structurale, IPBS, Université de Toulouse, Centre National de la Recherche Scientifique (CNRS), Université Paul Sabatier (UPS), Toulouse, France; ^6^ Ghent, Belgium Laboratory of Biochemistry, Department of Veterinary and Biosciences, Faculty of Veterinary Medicine, Ghent University, Gent, Belgium

**Keywords:** innate immunity, inflammation, macrophages, immune training, β-glucans, saccharomyces cerevisiae, escherichia coli, nutritional immuology

## Abstract

**Introduction:**

Confronted with the emerging threat of antimicrobial resistance, the development of alternative strategies to limit the use of antibiotics or potentiate their effect through synergy with the immune system is urgently needed. Many natural or synthetic biological response modifiers have been investigated in this context. Among them, β-glucans, a type of soluble or insoluble polysaccharide composed of a linear or branched string of glucose molecules produced by various cereals, bacteria, algae, and inferior (yeast) and superior fungi (mushrooms) have garnered interest in the scientific community, with not less than 10,000 publications over the last two decades. Various biological activities of β-glucans have been reported, such as anticancer, antidiabetic and immune-modulating effects. In vitro, yeast β-glucans are known to markedly increase cytokine secretion of monocytes/macrophages during a secondary challenge, a phenomenon called immune training.

**Methods:**

Here, we orally delivered β-glucans derived from the yeast S. cerevisiae to mice that were further challenged with Escherichia coli,

**Results:**

β-glucan supplementation protected the mice from E. coli intraperitoneal and intra-mammary infections, as shown by a lower bacterial burden and greatly diminished tissue damage. Surprisingly, this was not associated with an increased local immune response. In addition, granulocyte recruitment was transient and limited, as well as local cytokine secretion, arguing for faster resolution of the inflammatory response. Furthermore, ex-vivo evaluation of monocytes/macrophages isolated or differentiated from β-glucan-supplemented mice showed these cells to lack a trained response versus those from control mice.

**Conclusion:**

In conclusion, dietary β-glucans can improve the outcome of Escherichia coli infections and dampen tissue damages associated to excessive inflammatory response. The mechanisms associated with such protection are not necessarily linked to immune system hyper-activation or immune training.

## Introduction

The extensive use of antibiotics to treat bacterial infections in both humans and veterinary species has considerably accelerated the emergence of newly resistant bacterial strains. As stated by the World Health Organization, antimicrobial resistance (AMR) is rising to dangerously high levels in all parts of the world (1). In 2019, the Centers for Disease Control and Prevention (CDC) reported that more than 2.8 million antibiotic-resistant infections occur in the U.S. each year and more than 35,000 people die as a result (2). Among the many recommendations to prevent or slow the emergence of AMR, a reduction in antibiotic use in the agriculture sector is imperative. For example, among cattle, udder (mastitis) or respiratory tract infections by various bacteria are a major cause of antibiotic use (3–8). Consequently, the design of new therapeutic strategies that rely on new vaccine development and the identification of immune modulating molecules to synergize with antibiotics and help in controlling bacterial infections are of central importance. Among known biological response modifiers, β-glucans have been the focus of much interest. Indeed, β-glucans have been used and studied for their biological properties, including their immune-modulating properties, for decades (9). β-glucans are glucose polymers of which the structure is directly linked to their biological source. Cereal β-glucans consist of linear β- (1,3), β- (1,4) glucose polymers, whereas those from bacteria are linear with exclusively β-1,3 linkage and those from fungi exhibit side chains with β- (1,6) branching (10). Cereal β-glucan intake is associated with a prebiotic effect and various beneficial metabolic outcomes in hypercholesterolemia, diabetes, cardiovascular diseases, and hypertension (11), but does not appear to be associated with immune-modulation. On the contrary, a vast body of literature shows that fungi β-glucans modulate immune system activity and consequently interfere with infections, inflammation, and cancer progression (12, 13). Furthermore, the solubility, molecular mass, tertiary structure, and degree of branching can vary among β-glucans, leading to alterations of their immune-modulating effects. The manufacturing process and isolation method also influences the structure of β-glucans, explaining why β-glucans from the same source can trigger opposite cellular responses, even more so when the purity levels are not determined (reviewed in (14)). The immuno-modulating potential of β-glucans is attributed to their ability to prime and activate leucocytes through dectin-1, their main receptor expressed by macrophages, monocytes, dendritic cells, neutrophils, and a subset of T cells but not NK cells (15, 16). This feature has been extensively studied *in vitro* using various cellular models and, to a lesser extent, *in vivo* (17). However, the extensive diversity of β-glucan sources (reviewed in (18), molecular weight (solubility), tertiary structure (branching), and purity has hampered the elucidation of a prototypical mechanism through which β-glucans confer host protection against microbial infections. Although significant progress has been recently made in understanding the systemic effects of yeast β-glucans when administered by the parenteral route, the efficiency of orally administrated β-glucans is still debated or poorly understood (19, 20). However, the oral route is still probably the most convenient and innocuous way to deliver β-glucans to humans and animals (21).

In this context, our main objective was to determine whether oral supplementation with insoluble β (1,3)- (1,6)-glucans purified from the yeast *Saccharomyces cerevisiae* can confer protection to mice experimentally challenged with pathogenic *E. coli* strains in two infection models: peritonitis and mastitis. In parallel, we investigated local immune parameters resulting from the infection and its associated inflammatory response. Finally, through isolation/differentiation procedures, followed by *ex-vivo* secondary stimulation, we evaluated the possible link between β-glucan consumption and the pre-activation of monocyte/macrophage populations in the organism. Using this experimental setting, we found that oral β-glucans considerably prevented tissue damage and improved both infection outcome and resolution of the inflammatory response. Surprisingly, these benefits did not appear to arise from increased reactivity of monocyte/macrophage populations.

## Materials and methods

### Mouse strains and ethics statement

Wildtype C57Bl/6 mice were purchased from Janvier Labs (St Berthevin, France) and *Clec7a* ^-/-^ mice (22) were originally provided by Pr. Gordon Brown (University of Aberdeen, Scotland) and bred in-house. Eight week-old female C57Bl/6 *Clec7a* ^-/-^ mice and their strain-matched wildtype controls were housed under pathogen-free conditions in an accredited research animal facility of the National Veterinary College (Toulouse, France), which is fully staffed with trained husbandry, technical, and veterinary personnel. This study was carried out in strict accordance with the Federation of European Laboratory Animal Science Association guidelines (FELASA) and the local ethics committee ‘Science et Santé Animale’ (SSA) recommendations. Experiments were performed by FELASA-accredited investigators (n° 311155580). The intra-mammary experiments were performed at the Ghent University and were carried out in strict accordance with the recommendations in the Guide for the Care and Use of Laboratory Animals of the National Institutes of Health. The protocol was approved by the Committee on the Ethics of Animal Experiments of the Ghent University. Upon arrival in the facilities, mice were randomized. To avoid risk of esophagus lesions and minimize pain possibly induced by daily repeated force feeding with β-glucans, flexible end cannulas were used. Mice were euthanized by cervical dislocation.

### Preparation and oral administration of β-glucans

β-glucans were purified from *Saccharomyces cerevisiae* (*Sc*) (Strain owned by Lesaffre, France), as previously described (23). Briefly, the Sc wall was subjected to hot alkaline extraction (4% NaOH, 90°C, 2 h) to remove mannoproteins and lipids and was then lyophilized (94% dry matter). β-glucan 1,3/1,6 purity was determined using the enzymatic yeast β-glucan kit (K-EBHLG, Megazyme, Ireland) and the total amount of glucose released was measured by HPLC. The preparation was suspended in sterile D-PBS and delivered orally using dosing cannula at a dose of 0.15 mg/g of mouse weight every day for 14 days. Food and water were provided ad libitum for the duration of the experiments. The sham-dosed group of mice received the same volume (200 µL) of sterile D-PBS with a similar time schedule. After this force-feeding period, a three day rest period was observed before experimental challenge.

### Intraperitoneal infection model

*Escherichia coli* strain SP15, an extra-intestinal pathogenic *E. coli* strain (ExPEC) of serotype O18:K1:H7 isolated from a patient with neonatal meningitis (24), was provided by the laboratory of Pr. E. Oswald (IRSD, Toulouse, France). SP15 was cultured overnight at 37°C with rotary shaking (200 rpm) in LB medium. Sub-cultures were initiated by diluting an overnight culture by 1/100 in LB and further growth for 3 h. Optical density was measured at 600 nm and the concentration adjusted to 1x10^8^ bacteria/mL. The number of colony-forming units (CFUs) was determined by plating serial dilutions on LB agar. One million (1x10^6^) CFUs diluted in sterile D-PBS were injected per mouse in the abdominal right lower quadrant (400 µL). Mice were euthanized at the indicated time points after challenge and biological samples collected. First, the peritoneal cavity was washed with 5 mL sterile D-PBS containing 2 mM EDTA and 5% heat-inactivated fetal calf serum (FCS) to collect the exudates. Peritoneal exudates were then centrifuged (300 x g, 4°C), the peritoneal cell pellets processed for flow cytometry analysis, and the supernatants stored at -80°C for cytokine quantification. The liver and spleen of each mouse were collected. For each organ, a piece of tissue was fixed in 10% formalin solution for histological examination by a pathologist in a blind fashion. Another piece of tissue was mechanically dissociated (ribolysis) for CFU determination by serial dilution of the homogenates and plating on MacConkey agar. One femur per mouse was dissected, the epiphysis cut away, and the medulla flushed with a 26-gauge needle mounted on a syringe. Myeloid cells were pelleted and red blood cells lysed using a dedicated solution (Miltenyi Biotec, Germany). Fixed tissues were embedded in paraffin and subjected to standard hematoxylin and eosin staining.

### Intramammary infection model

*E. coli* strain P4 (O32:H37) was isolated from a cow with clinical mastitis and is now considered to be a reference strain (25). For intramammary inoculation, P4 expressing mCherry was used. One day prior to inoculation, bacteria were grown 5 h at 37°C in brain heart infusion (BHI) medium under agitation. A 1/100 dilution of the preculture was further grown in BHI overnight at 37°C. After centrifugation and two washing steps, bacteria were suspended and diluted in sterile PBS and then adjusted to 1x10^4^ CFU/mL using a standard curve plotting CFUs as a function of absorbance at 600 nm. The number of CFUs actually injected was confirmed by plating the inoculum onto an agar plate and counting the CFUs after overnight incubation. Eight-week-old C57BL/6 female mice were coupled with 10-week-old males. Following parturition, the pups were weaned after 10 days to enhance mammary gland development. One hour after weaning, mice were intramammarily injected with the bacterial suspension or PBS in the fourth gland pair (R4 and L4) under inhalational anesthesia. Briefly, the teats and the surrounding area were disinfected with a sterile compress soaked in a chlorhexidine solution. While lightly maintaining the teat with forceps near the apex, a 32-gauge blunt needle mounted on a syringe was gently inserted through the canal and the corresponding volume slowly delivered. Twenty-four hours after bacterial challenge, mice were euthanized by cervical dislocation and the inoculated glands isolated and prepared for *ex-vivo* imaging before mammary sampling for histological examination and digestion for flow cytometry analysis.

### *Ex-vivo* bioimaging

*Ex-vivo* imaging was performed using an IVIS lumina II device (Caliper). The bacterial load was quantified by measuring mCherry fluorescence emission at 610 nm. Neutrophil influx was detected by measuring MPO activity after Luminol injection into the gland. A stock solution of Luminol (10 mg/mL) was prepared before injection into the mammary glands (100 mg/kg) under isoflurane-anesthesia. After 2 min of incubation, the mammary glands were dissected and both fluorescence and bioluminescence signals were acquired. Luminescence data are provided as the average radiance expressed in photons/second/cm²/sr, which represents the photon emission from the tissue surface, and the fluorescence results are expressed as mean MFI.

### Isolation of mammary leukocytes

Immediately after imaging, lymph nodes were carefully removed from the mammary glands (L4 and R4) and the mammary glands immediately placed in cold PBS. The glands were weighed and homogenized using a McIlwain chopper. Tissue homogenates were suspended in 0.1% type IV collagenase freshly diluted in RPMI and then incubated for 30 min at 37°C under agitation. Cells were then pelleted, suspended in 40% Percoll, layered onto 80% Percoll, and finally centrifuged at 1,000 x g for 30 min at 15°C. The gradient interface, containing leukocytes, was collected and washed in 5% FCS-supplemented PBS and finally suspended in cold 0.2 µm-filtered FACS buffer containing PBS, 1% bovine serum albumin (BSA), 2.5 mM EDTA, and 0.01% sodium azide. The number of isolated leukocytes and dead cells was determined by flow cytometry. For further analysis, 2x10^5^ cells per gland were fixed in a 4% paraformaldehyde (PFA) solution in PBS for 10 min. After PBS washing steps, leucocytes were stored in PBS at 4°C until analysis.

### Flow cytometry analysis

Before staining, cells were incubated with FcBlock reagent (anti-CD16/CD32, Biolegend, Ozyme-France) to block the FcγRII/III receptors. The cellular content of the peritoneal exudates was characterized using a combination of fluorochrome conjugated mAbs: CD45-vioblue (REA737, Miltenyi Biotec), Ly6G-FITC (1A8, Miltenyi Biotec) F4/80-APC (CI:A3-1; AbD Serotec, BioRad, Luxembourg), Ly6C-PE (HK1.4, Biolegend), and CCR3-APC-Fire (J073E5, Biolegend). At analysis, doublets and dead cells, labeled with 7-AAD (Biolegend), were excluded. All samples were acquired using a MACS Analyzer (Miltenyi Biotec) flow cytometer and analyzed using FlowJo software (TreeStar, USA). The gating strategies are presented in Supplementary Material. Mammary gland cells were labeled with the following fluorochrome conjugated mAbs: anti-CD45-PECy7 (I3/2.3; Southern Biotech), Anti-Ly6G-FITC (1A8, Miltenyi Biotec), and anti-F4/80-APC (CI:A3-1; AbD Serotec) to identify leukocytes, neutrophils, and macrophages, respectively. At analysis, doublets, red blood cells, and lymphocytes were excluded based on CD45 staining. All samples were acquired using a CytoFLEX (Beckman Coulter, USA) flow cytometer and analyzed using CytExpert software (Beckman Coulter, USA).

### Macrophage isolation and differentiation from bone-marrow precursors

Mice were supplemented with β-glycans or PBS as control, as described in the corresponding section. At the time of euthanasia, peritoneal cells were harvested as described above and the femurs dissected to collect bone marrow. Resident peritoneal macrophages (RPMs) were isolated at steady state from wildtype or dectin-1 deficient C57BL/6 mice. RPMs were sorted by positive magnetic cell sorting (MACS^®^, Miltenyi Biotec) using Anti-F4/80 MicroBeads UltraPure (Ref: 130-110-443, Miltenyi Biotec) serially applied on two individual MS columns. Purity and viability were assessed by flow cytometry, and each was > 95%. Bone-marrow-derived macrophages (BMDMs) were prepared as previously described (26) after culture with mouse recombinant M-CSF (25 µg/mL) for six days. BMDMs were collected in ice-cold non-enzymatic PBS-EDTA (5 mM) buffer. Bone-marrow monocytes (BMMs) were isolated from β-glucan- or PBS-supplemented wildtype C57BL/6 mice and sorted by negative selection using a Monocyte Isolation Kit (Bone Marrow) (Ref: 130-100-629, Miltenyi Biotec), as recommended by the manufacturer. BMM purity was > 90% and viability > 95%. For each BMM preparation, two mice were pooled to reach sufficient cell numbers. RPMs, BMDMs, and BMMs were seeded at 10^5^ cells in 96-well culture plates in complete RPMI Glutamax II (10% FCS, 1% Sodium pyruvate, 1% penicillin streptomycin) and left to adhere for 2 h. Cultures were then stimulated overnight with 100 ng/mL ultrapure LPS from *E. coli* (0111:B4, *In vivo*gen, France). Supernatants were collected and stored at -80°C for TNF-α and IL-6 measurement.

### Cytokine secretion measurement

Cytokine production was quantified using a customized multiplex (Milliplex-MAP, Merck Millipore, France) assay kit and Luminex 100 IS instrument (Luminex, USA) available at the phenotyping service platform Anexplo (CHU Rangeuil, Toulouse, France). Ten cytokines were systematically assayed in peritoneal exudates: IL-1α, IL-1β, IL-6, CXCL1, CXCL2, CXCL5, CCL2, CCL5, BAFF, and TNF-α. For BMDM culture supernatants, individual cytokine-detection kits were used to quantify mouse IL-6, and TNF-α (Biolegend, Ozyme-France).

### Statistical analysis

Individual experiments and the number of mice used in each experiment are provided in **Supplementary Table 1**. Statistical computing was performed and graphs generated using R software. Graphs were generated using the ggplot2 R package (27). Cell numbers/percentages or cytokine concentrations were compared between the two experimental groups at the indicated time points using Welch’s t-test, an adaptation of Student’s t-test for groups of unequal variance and/or size. When significant (p < 0.05) or near significance, raw p-values are directly indicated on the graphs. Heatmaps were computed after transforming the cytokine concentration into Z-scores and generated using the Pheatmap R package (28). Multiple factor analysis (MFA) and hierarchical clustering were performed using the FactoMiner R package (29) after Z-score transformation of the quantitative variables. Luminescence and fluorescence intensities from ex-vivo live imaging experiments were compared by one-way ANOVA followed by a pair-wise Wilcoxon test.

## Results

### β-glucan supplementation reduces bacterial fitness and alleviates tissue damage after *E. coli* i.p. challenge

**Figure 1:**
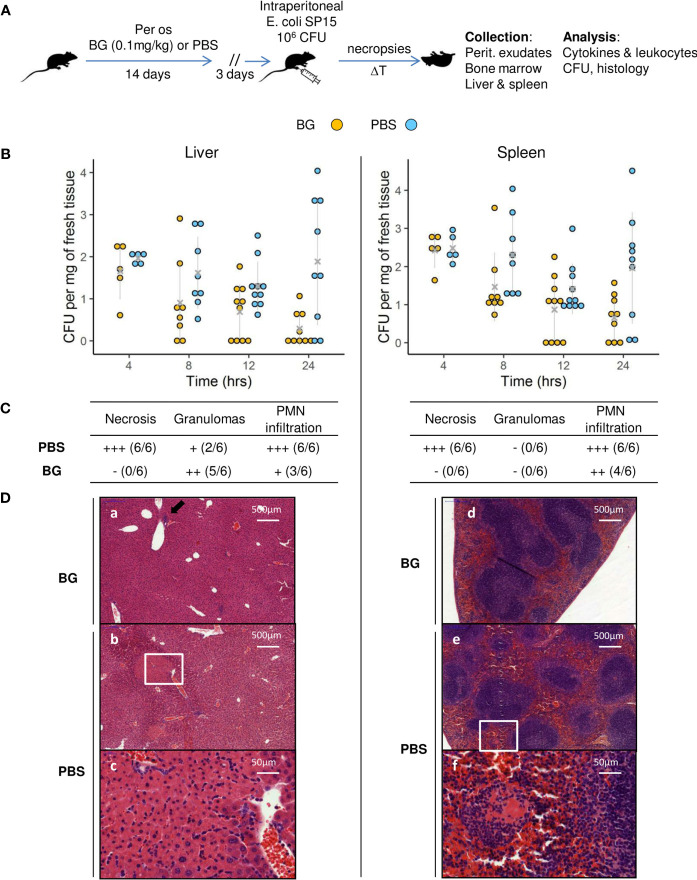
Bacterial fitness and the host response in liver and spleen and histological examination after *E*.*coli* intra-peritoneal challenge of β-glucan or PBS orally-supplemented C57BL/6 mice. **(A)** Experimental design. **(B)** Bacterial loads in log CFUs per mg of fresh tissue in the liver and spleen over time. Grey crosses and bars represent the mean ± SD. Statistical analysis was performed using the Welch t-test and significant p values are indicated. **(C)** Histological examination of hematoxylin eosin-stained sections of liver and spleen sampled at 24 h p.i. Magnified areas are indicated by white delimitations. **(D)** Results of the semi-quantitative grading of histological features (–) absent, (+) minimal, (++) moderate, and (+++) severe and the number of mice from each group presenting these features. The number of mice used and experiments are detailed in **Supplementary Table 1**.

After two weeks of oral supplementation with β-glucans or PBS only, mice were infected with *E. coli* and sequentially euthanized to assess both their infectious and immune parameters (**Figure 1A** and **Supplementary table 1**). Of note, no mouse died during the follow-up, regardless of the experimental group. To evaluate bacterial fitness, the number of colony-forming units (CFUs) was determined at necropsies in the liver and spleen (**Figure 1B**), two organs in which i.p. bacterial infections frequently disseminate. Mice that received PBS only showed CFU values that were stable over time for both the liver and spleen. However, at 24 h p.i., the CFU values were heterogeneous, showing that some mice had cleared the infection (liver 2/9, spleen 2/9), whereas others showed high CFU values. However, the mean CFU values recorded 4 h p.i. were not statistically different from those recorded at 24 h p.i. The results for mice supplemented with β-glucans were highly different. Indeed, there was a negative correlation between the time since inoculation and the CFU values and comparison of the mean number of CFU between 4 and 24 h showed a significant decrease (p = 0.0034). Moreover, a sizable number of mice cleared the infection as soon as 12 h p.i. (12 h: liver and spleen 4/10, 24 h: liver 5/9, spleen 3/9). Consequently, the mean number of CFUs in the β-glucan group was statistically lower than that in the PBS group at 12 and 24 h p.i. in the liver and at 24 h p.i. in the spleen (p = 0.019, p = 0.006, and p = 0.016 respectively). Overall, these results show that β-glucan supplementation reduced bacterial fitness relative to the control mice, in which bacterial growth appeared to be poorly controlled. Tissue damage is a good indicator of the severity of infection. The grading of histological alterations (24 h p.i.) showed the liver and spleen of PBS mice to be severely damaged, with large necrotic areas, and intense recruitment of neutrophils, consistent with a higher number of CFUs and, possibly, a strong inflammatory reaction (**Figure 1C**). Livers from control mice showed large areas of hepatocyte lysis, with two types of necrosis. First, all mice showed large areas of ischemic-like necrosis in the perivascular spaces, with little or no neutrophilic infiltration. Such necrosis was characterized by swollen hepatocytes showing an eosinophilic cytoplasm [**Figure 1D**, panels (b) and (c)]. In the cortical sinus, we observed massive areas of necrosis dominated by intense recruitment of neutrophils (data not shown). By contrast, livers from mice supplemented with β-glucans showed no necrosis [**Figure 1D**, panel (a)] and limited numbers of neutrophils grouped within small granulomas in proximity to the perivascular spaces [**Figure 1D** (a), black arrow]. Similarly, the spleens from PBS mice were also highly damaged by necrosis due to massive neutrophil infiltration [**Figure 1D**, panel (e)], which was not observed in β-glucan mice. Of note, lymphocyte activation, reflected by the number of distinguishable clear centers, appeared to be lower in mice from the PBS group [**Figure 1D**, panels (d) and (e)]. Oral supplementation with β-glucan protected the liver and spleen from tissue damage, suggesting better control of the infection and/or better regulation of inflammatory processes.

### β-glucan-supplemented mice show significantly different inflammatory responses to *E. coli* i.p. challenge than those given PBS

**Figure 2:**
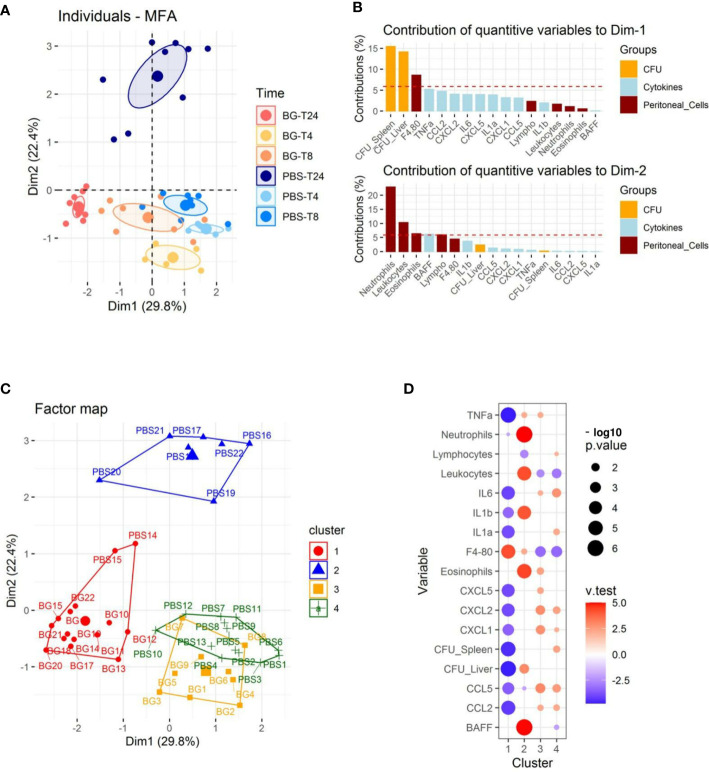
Multifactorial analysis and hierarchical clustering of immune and bacteriological parameters recorded from individual mice sampled at 4, 8, and 24 h p.i. **(A)** Individual factor map. **(B)** Respective contribution of quantitative variables to dimensions 1 and 2. **(C)** Cluster plot from hierarchical clustering performed according to Ward’s Method. **(D)** Bubble plot indicating variables positively (red) or negatively (blue) characterizing clusters according to the test value (v-Test). Bubble sizes reflect the statistical significance (-log10 p value). The number of mice used and experiments are detailed in **Supplementary Table 1**.

We performed multifactorial analysis (MFA) using immune-response and bacteriological data (cell recruitment and cytokine release in peritoneal exudates, CFU values) and the time post-infection (4, 8, and 24 h p.i.) to identify the parameters that segregate the mice (**Supplementary Table 1**). The first two dimensions accounted for 52.2% of the total variance of the dataset (**Figure 2A** and **Supplementary Figure 1A**). The first axis (Dim1) compares mice with a high number of CFUs to those with a low bacterial burden (**Figure 2B** and **Supplementary Figure 1B**), which correlated with the time post-infection (**Figure 2A**). Indeed, mice sampled at later time points (24 h p.i.) had fewer CFUs, which was particularly true for mice supplemented with β-glucans. The second axis (Dim2) compares mice showing reduced peritoneal cell recruitment to those with high numbers of leukocytes, mainly granulocytes, present in their peritoneal exudates. At earlier time points (4 and 8 h p.i.), mice from the β-glucan and PBS groups were poorly separated by Dim1 and Dim2. By contrast, at 24 h p.i., the two experimental groups were highly separated in both dimensions, in which mice from the PBS group were characterized by a higher number of CFUs and greater granulocyte recruitment than those from the β-glucan group, which showed limited bacterial fitness and lower recruitment of granulocytes. Cytokine secretion also contributed to Dim1 but to a lesser extent (**Figure 2B**). We refined this preliminary step of analysis by clustering the mice according to Ward’s Method using the same dataset. Four main clusters were found using this approach (**Figure 2C**). The first was mainly composed of mice from the β-glucan group (all mice sampled at 24 h and 4 mice at 8 h) and two mice from the PBS group sampled at 24 h p.i. Inversely, cluster 2 exclusively included control mice sampled at 24 h p.i. This confirmed the preliminary visualization of individuals by MFA (**Figure 2A**). The third cluster was comprised of only mice from the β-glucan group (all mice sampled at 4 h p.i. and 5 mice sampled at 8 h p.i.). The last cluster included all control mice sampled at 4 and 8 h p.i. This clustering confirmed that the β-glucan and PBS groups mainly differed at the later time points of the infection follow-up. The contribution of each recorded variable to the clusters is presented as a bubble plot chart (**Figure 2D**). Thus, compared to the mice of the other clusters, cluster 1 mice were characterized by significantly less inflammatory cytokine secretion and a lower number of CFUs in the liver and spleen but higher infiltration of F4-80-positive cells. By contrast, cluster 2 mice were associated with greater leukocyte recruitment, with numerous neutrophils and eosinophils, a higher number of CFUs in the liver, and the secretion of BAFF, an inflammatory cytokine produced by innate immune cells, including neutrophils (30). As expected, due to their overlapping, clusters 3 and 4 shared highly similar features. Mice from these clusters showed higher inflammatory cytokine levels but lower leukocyte recruitment than those of clusters 1 and 2, mainly due to less F4-80^pos^ cell recruitment. Overall, oral supplementation with β-glucans led to substantial modifications of the inflammatory response. Notably, both cytokine secretion and cell recruitment were differentially regulated, especially at later time points of the infection.

### β-glucan supplementation leads to earlier regulation of inflammatory cytokine secretion

**Figure 3:**
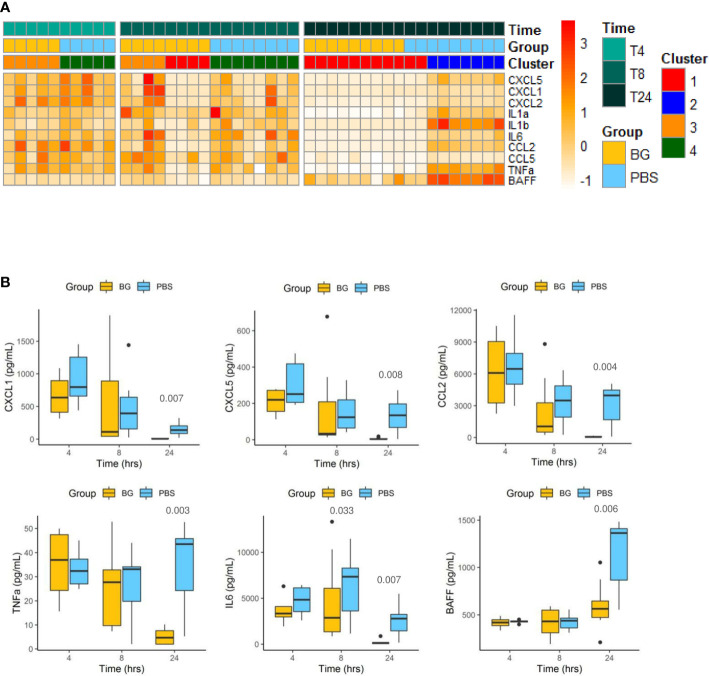
Cytokine secretion in peritoneal exudates from individual mice at 4, 8, and 24 h after challenge. **(A)** Heatmap representation of the cytokine concentrations of each individual (after Z-score transformation) according to experimental group, cluster, and sampling time point. **(B)** Cytokine concentrations (pg/mL) in exudates determined by multiplexed ELISA. Statistical analysis was performed using the Welch t-test and significant p values are indicated. The number of mice and experiments are detailed in **Supplementary Table 1**.

We extended the analysis by assessing cytokine secretion in peritoneal exudates during the course of the E. coli infection in both groups. Cytokine concentrations are first presented as a heatmap according to group, time post infection, and clustering (**Figure 3A**). At 4 h p.i., the cytokine secretion patterns were highly similar between the PBS and β-glucan-supplemented groups and between individuals included in clusters 3 and 4. This time point represented the peak of secretion for most of the cytokines analyzed, except BAFF, which showed a different secretion pattern. At 8 h p.i., all mice from the PBS group were still secreting cytokines, but half of the β-glucan-supplemented mice showed a strong reduction in cytokine secretion in the peritoneal exudates. These animals corresponded to those included in cluster 1 as opposed to cluster 3. This tendency was strikingly confirmed at 24 h p.i., at which time all the mice in the β-glucan group formed cluster 1, and, consequently, all mice in this group had arrested cytokine secretion. By contrast, most PBS mice (cluster 2) still showed relatively high cytokine secretion. This was further confirmed by individual analysis of each cytokine secretion pattern. For CC and CXC chemokines, the secretion pattern reflected a progressive decrease from 4 to 24 h p.i., but this decrease was strongly accentuated in the β-glucan-supplemented mice (**Figure 3B** and **Supplementary Figure 2**). TNFα secretion remained constant in PBS mice, whereas it strongly decreased between 8 and 24 h p.i. in the β-glucan-supplemented animals. Similar observations were made for IL-6. Inversely, BAFF progressively accumulated in peritoneal exudates according to the intensity of the inflammation. BAFF sharply increased between 8 and 24 h p.i. in the PBS group, whereas it merely rose above basal levels in the β-glucan mice. Overall, these results show that β-glucan supplementation resulted in more precocious regulation of cytokine secretion and suggest major consequences on inflammatory cell recruitment and differentiation.

### β-glucan supplementation alters both the kinetics and intensity of leukocyte recruitment

**Figure 4:**
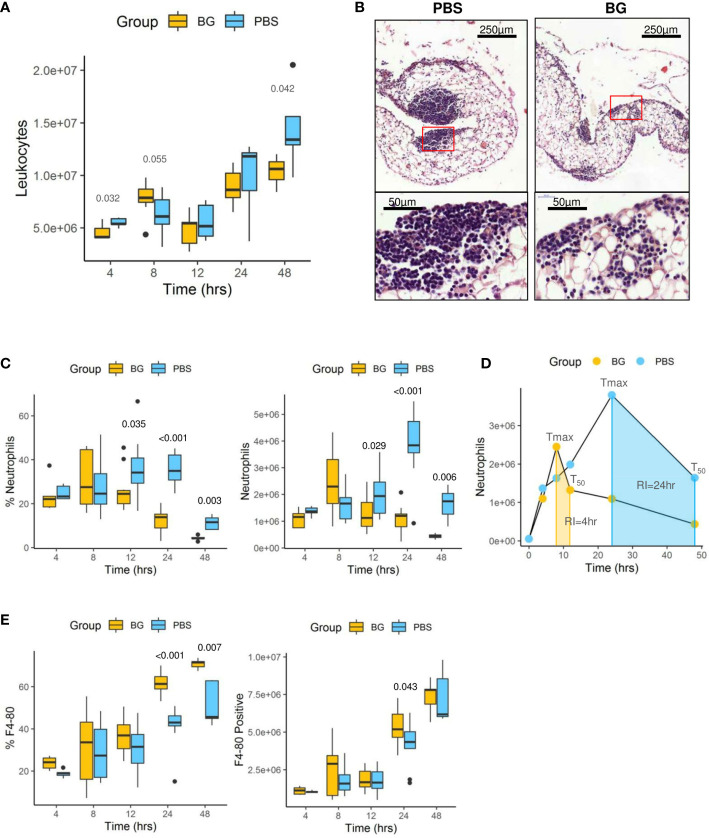
Leukocyte infiltration in the peritoneal cavity. **(A)** Flow-cytometry assessment of CD45^+^ leukocyte numbers. **(B)** Histological examination of hematoxylin eosin-stained sections of omentum sampled at 24 h p.i. **(C)** Flow-cytometry assessment of neutrophil (Ly6G^pos^) frequency and number in peritoneal exudates. **(D)** Neutrophil dynamics in each experimental group (T_max_: time for maximal neutrophil recruitment, T_50_: time for neutrophil numbers to decrease to half T_max_, RI: resolution interval = T_max_ – T_50_). **(E)** Flow-cytometry assessment of F4-80^pos^ cell frequencies and numbers in peritoneal exudates. Statistical analysis was performed using the Welch t-test and significant p values are indicated. The number of mice used and experiments are detailed in **Supplementary Table 1**.

The total number of CD45^pos^ leukocytes was determined in the peritoneal exudates from 4 h up to 48 h after *E. coli* infection (**Figure 4A**). Leukocyte accumulation was progressive and biphasic. A first wave of accumulation occurred between 4 and 12 h p.i. and a second wave between 12 and 48 h p.i. There were no differences in the total number of CD45^pos^ leukocytes between experimental groups. We further investigated leukocyte recruitment by histological examination of the omentum at 24 h p.i. In control mice, we observed massive aggregates of immune cells, also called milky spots, in the omentum, whereas milky spots from β-glucan-supplemented mice were much smaller and less numerous (**Figure 4B**).

**Figure 5:**
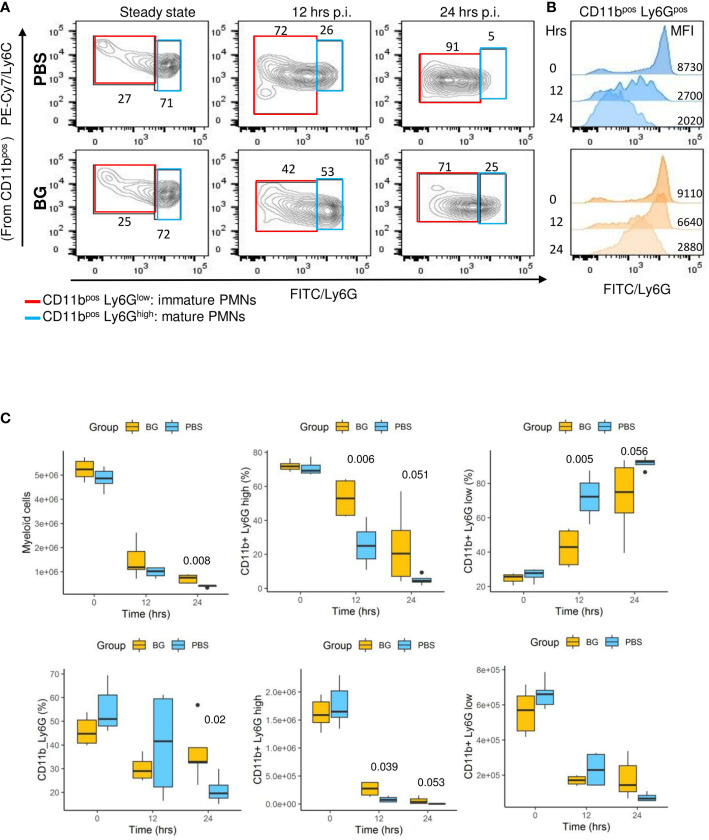
Neutrophil differentiation in bone marrow after oral supplementation and **(*E*)** *coli* challenge. **(A)** Flow-cytometry analysis of myeloid CD11^pos^, Ly6G^High^, or Ly6G^Low^ cell frequencies in bone marrow over time (the frequencies indicated are representative of the mean frequencies recorded for each group at each time point). **(B)** Flow-cytometry assessment of Ly6G mean fluorescence intensity among CD11b^pos^ cells. **(C)** Flow-cytometry determination of CD11b^pos^, CD11b^pos^Ly6G^high^, or Ly6G^low^ frequencies and numbers in bone marrow at steady state and after *E*. *coli* challenge. Statistical analysis was performed using the Welch t-test and significant p values are indicated. The number of mice and experiments are detailed in **Supplementary Table 1**.

Flow cytometry analysis (gating strategy presented in **Supplementary Figure 3**) of peritoneal exudates showed progressive accumulation of polymorphonuclear neutrophils (PMNs) (**Figure 4C**) and eosinophils (**Supplementary Figure 4A**), but with different recruitment kinetics for each experimental group. Indeed, in the β-glucan group, PMN accumulation peaked at 8 h p.i. and then progressively decreased to return to basal levels at 48 h p.i. By contrast, PMN recruitment in PBS mice appeared to be much slower and peaked at 24 h p.i., although the peak intensity was much greater in this case. The dynamics of neutrophil recruitment can be used to evaluate the resolution of inflammation by determining a resolution index (RI) calculated from the time of the PMN peak (T_max_) to when it has decreased by half (T_50_) (31). The β-glucan group showed a RI of 4 h versus that of the PBS group that showed a markedly extended RI of 24 h (**Figure 4D**). These observations show that β-glucan supplementation not only altered cytokine secretion but also modified the dynamics of PMN recruitment and consequently the inflammatory reaction to achieve earlier regulation. Moreover, the above results, as suggested by the MFA, suggest that β-glucan supplementation may have consequences on F4-80^pos^ cell recruitment. This labeling strategy (**Supplementary Figure 3**) only allowed us to confirm that the proportion of F4-80^pos^ in exudates was significantly different between the experimental groups at later time points (**Figure 4E**) but was insufficient to determine whether it concerned resident or recruited mononuclear cells. The lymphocyte dynamics in the peritoneal exudates were very similar for both groups. However, at 4 h p.i., the lymphocyte numbers in the β-glucan mice were significantly lower (**Supplementary Figure 4B**), suggesting a more precocious inflammatory reaction. As highly different kinetics of granulocyte recruitment were recorded in the peritoneal exudates, the neutrophil differentiation stage was analyzed by flow cytometry in the bone marrow (**Supplementary Figure 5**). At steady state, the total number of CD11b^pos^ myeloid cells was similar in the two experimental groups. Similarly, there were no differences in the percentage or absolute numbers of CD11^pos^ Ly6G^pos^ PMNs and the proportion of mature (70% Ly6G^high^) and immature (30% Ly6G^low^) PMNs was unaffected by supplementation (**Figure 5A and C**). The peritoneal challenge with *E. coli*. induced the depletion of myeloid cells from 12 h p.i., including PMNs, in both groups. At 24 h p.i., PBS mice showed severe medullar aplasia, consistent with the peripheral consumption of leukocytes (**Figure 5C**). Moreover, a striking decrease in the frequency of mature PMNs was observed in the PBS group as soon as 12 h p.i. (**Figure 5A**), as shown by a sharp decrease in Ly6G mean fluorescence intensity (MFI) (**Figure 5B**). We did not observe such a decrease in β-glucan-supplemented mice, which conserved significantly higher numbers of mature PMNs throughout the infection. Despite a precocious peak of recruitment in the peritoneal cavity, β-glucan mice conserved a higher frequency of mature Ly6G^high^ neutrophils than control mice (**Figure 5**). Given the absence of a difference between the two groups at steady state, this observation suggests that the peripheral consumption of PMNs in β-glucan mice was limited or that the replenishment of bone-marrow PMN stocks was more efficient.

### β-glucans supplementation increases monocyte recruitment and accelerates their differentiation after *E. coli* i.p. challenge

**Figure 6:**
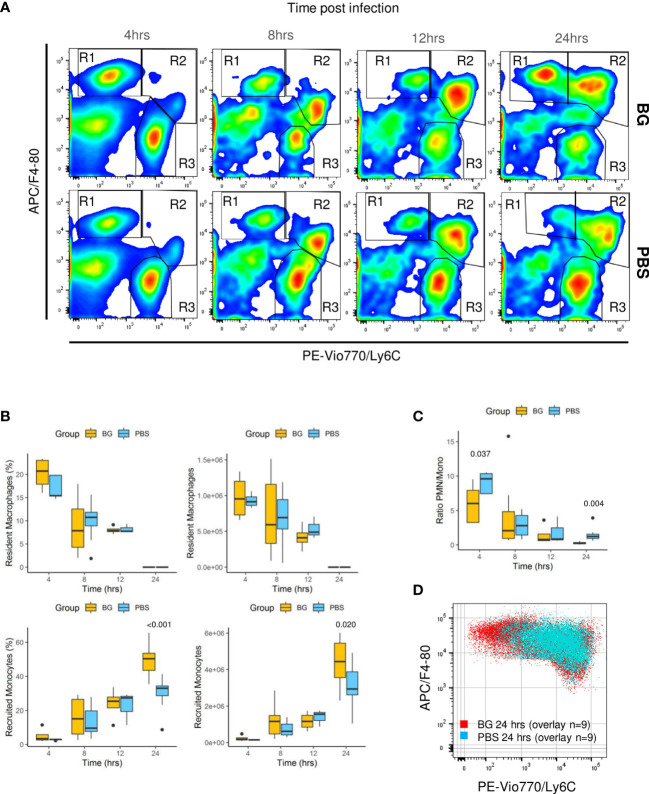
Flow-cytometry analysis of F4-80^+^ subpopulations from peritoneal exudates collected throughout the infection. **(A)** Representative flow-cytometry panels of F4-80^pos^/Ly6C^pos^ cell distributions over time in each experimental group. **(B)** Frequencies and numbers of F4-80^high^ Ly6C^low^ resident peritoneal macrophages or F4-80^low^ Ly6C^high^ recruited inflammatory monocytes. **(C)** Ratio of PMN over recruited monocyte numbers over time. **(D)** Overlay of F4-80^pos^/Ly6C cells from nine β-glucan-supplemented mice and nine PBS control mice at 24 h p.i. Statistical analysis was performed using the Welch t-test and significant p values are indicated. The number of mice and experiments are detailed in **Supplementary Table 1**.

As F4-80^pos^ cells include various cell types from resident macrophages to recruited inflammatory monocytes, which further differentiate into macrophages or dendritic cells, we conducted a more in-depth characterization of the cell types. Three main CCR3^neg^ cell populations were identified using Ly6C in combination with the F4-80 marker and CCR3 to exclude F4-80^pos^ eosinophils: F4-80^high^ Ly6C^neg^ (R1), considered to be resident peritoneal macrophages in the early steps of the infection; F4-80^low^ Ly6C^high^ (R2), considered to be recruited inflammatory monocytes; and F4-80^neg^, Ly6C^low^, considered to be PMNs (R3) (**Figure 6A**, **Supplementary Figure 6**). Analyzing the R3 gate over time confirmed that the number of PMNs was rapidly downregulated from 4 to 24 h p.i. in the β-glucan mice. By contrast, as previously observed with the specific Ly6G labeling, the PMNs numbers increased over time in the control mice (**Figure 6A**). Resident macrophages (percentage and numbers) from R1 progressively decreased as both the infection and inflammation developed, in accordance with current knowledge. Of note, there were no differences between experimental groups (**Figure 6B**). In parallel, inflammatory monocyte numbers increased (R2). However, at 24 h p.i., the cell population from the R1 gate had strikingly increased in the β-glucan group, whereas it continued to decrease in the PBS group. Although impossible to ascertain without cell tracing experiments, it was highly likely that infiltrated inflammatory monocytes started to differentiate into F4-80^high^ cells. Indeed, the R1- and R2-gated cells were clearly disconnected at the early time points (4 and 8 h p.i.), arguing that they were two distinct cell populations (**Figure 6A**). However, a continuum between the two gates formed progressively over time, strongly suggesting cell differentiation from R2 to R1. This process appeared to occur much more rapidly in the β-glucan-supplemented group. Consistent with these observations, overlapping dot plots from each individual mouse sampled 24 h p.i. confirmed that recruited monocytes from β-glucan mice differentiated more rapidly than those of control mice, based on upregulation of the F4-80 marker in parallel with Ly6C downregulation (**Figure 6D**). As a consequence, both the percentage and number of infiltrating/differentiating monocytes were significantly higher in β-glucan-supplemented mice (**Figure 6B**) and, coupled with faster downmodulation of the number of PMNs, led to a much lower PMN/monocyte ratio (**Figure 6C**). Overall, these results further emphasize that regulation of the inflammatory reaction was improved by β-glucan supplementation.

### β-glucan supplementation does not induce monocyte/macrophage immune training

**Figure 7:**
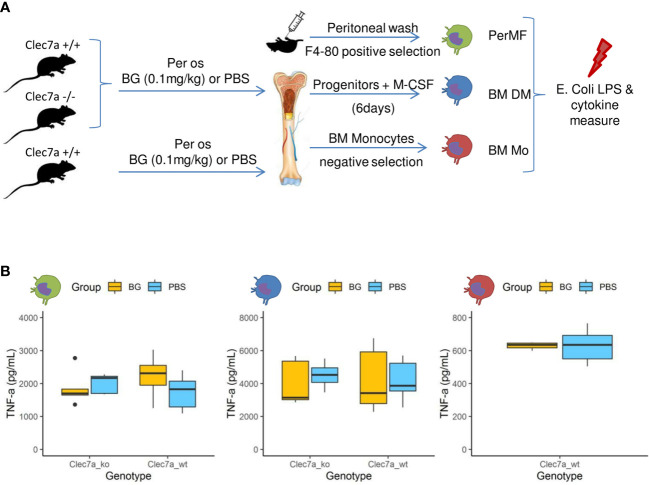
Cytokine secretion by macrophages or monocytes isolated or generated from β-glucan- or PBS-supplemented mice at steady state. **(A)** Experimental design. **(B)** TNFα (pg/mL) secretion in culture supernatants of (green) resident peritoneal macrophages, (blue) bone marrow-derived macrophages, or (red) bone-marrow monocytes stimulated for 24 h with *E*. *coli* LPS. Statistical analysis was performed using the Welch t-test and significant p values are indicated. For PerMF and BMDM, the data were collected from two individual experiments representing a total of eight wildtype and five *clec7a* ^-/-^ β-glucan^-^supplemented mice and 10 wildtype and five *clec7a* ^-/-^ PBS-supplemented mice. For monocytes isolated from bone marrow, the data were collected from a single experiment with a total of six wildtype β-glucan- and six PBS-supplemented mice pooled two by two to reach sufficient monocyte numbers.

A hallmark of β-glucan treatment is the ability to increase the secretion of inflammatory cytokines after secondary exposure to a microbe or its ligands as a consequence of epigenetic and metabolic reprogramming (32). We evaluated this possibility by feeding wildtype mice or mice deficient for the *Clec7a* gene, encoding dectin-1, the cognate receptor of β-glucans, β-glucans or PBS for two weeks. Then, resident peritoneal macrophages were isolated by magnetic positive selection (PerMF). In parallel, myeloid precursors were isolated and further differentiated into macrophages using M-CSF (BMDM). Both PerMF and BMDM were stimulated *ex vivo* with LPS and TNFα (or IL-6: **Supplementary Figure 7**) and cytokine secretion measured (**Figure 7A**). Under these settings, cytokine secretion was similar between the β-glucan-supplemented and PBS groups, with no influence of the genetic background (**Figure 7B**). To confirm this result, bone-marrow monocytes were isolated using a depletion-based sorting strategy and, as above, stimulated *ex vivo* with LPS. Again, β-glucan supplementation had no effect on cytokine secretion (**Figure 7B**).

These experiments show that the β-glucan-mediated upregulation of cytokine secretion by monocyte-derived populations usually observed *in vitro* did not occur in our experimental model. These results are consistent with the *in vivo* cytokine measurements from peritoneal exudates, which never indicated increased production in β-glucan-supplemented mice, regardless of the time considered post infection.

### β-glucan supplementation mitigates *E. coli* mammary gland infection

**Figure 8:**
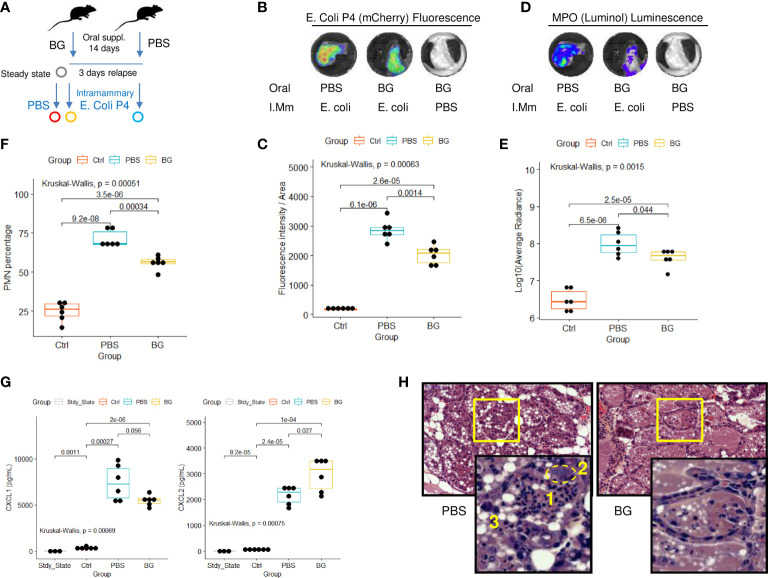
Intra-mammary infection with *E*. *coli* strain P4 in β-glucan- or PBS-supplemented mice. **(A)** Experimental design, for which the steady state corresponds to β-glucan mice euthanized after the supplementation period but with no intra-mammary injections (n = 3), Ctrl corresponds to β-glucan-supplemented mice that received a PBS injection in one mammary gland (n = 6 mice), β-glucan (n = 6) and PBS (n = 6) correspond to mice supplemented as indicated and infected intra-mammarily with *E. coli* P4. *Ex-vivo* imaging of **(B, C)** fluorescence or **(D, E)** luminescence intensities of dissected mammary glands. Images are representative of each experimental group. **(F)** Flow-cytometry analysis of neutrophil recruitment (CD45^pos^ Ly6G^pos^ cells) in mammary gland tissue. **(G)** CXCL1 and 2 chemokine levels measured by ELISA from mammary gland tissue homogenates. Statistical comparisons were performed using non-parametric Kruskal-Wallis tests followed by multiple comparison tests (Wilcoxon) corrected using the Bonferroni method (indicated p-values are adjusted). **(H)** Hematoxylin - eosin histological examination of mammary gland tissue from PBS or β-glucan-supplemented mice infected with *E*. *coli* P4 (large panel magnification x200, small panel x800) (1): neutrophil infiltration, (2) cluster of bacteria, (3) swelled necrotic alveolar cells.

As stated in the introduction, mammary gland infections in dairy cattle are a leading cause of antibiotic consumption and, among others, *E. coli* is commonly found to be responsible for such infections (33). Consequently, we designed a mammary gland infection model with mCherry fluorescent *E. coli* in mice supplemented with β-glucans or PBS (**Figure 8A**). *Ex-vivo* measurement of mCherry fluorescence in dissected glands showed those from β-glucan-supplemented mice to exhibit lower fluorescence intensities than those from PBS mice (**Figures 8B, C** and **Supplementary Figure 8A**). This demonstrates that, as previously observed during *E. coli* peritoneal challenge, β-glucan supplementation reduced bacterial fitness. In parallel, phagocyte (PMN/macrophage) recruitment was quantified by measuring myeloperoxidase (MPO) activity after injecting Luminol into the gland. MPO luminescence was significantly higher in the glands of PBS mice (**Figures 8D, E**). We confirmed this result and more precisely determined the cells involved in this signal by digesting the mammary gland tissue and analyzing the resulting cell suspension by flow cytometry. Again, paralleling the results using our peritonitis model, neutrophil recruitment was more intense in the glands from PBS mice than those from β-glucan-supplemented mice (**Figure 8F**). Cytokine levels in the mammary gland tissue were not affected by oral supplementation at steady state and remained below the detection threshold (**Figure 8G**). Few statistical differences were observed between the PBS and β-glucan experimental groups for most of the cytokines measured. However, PBS mice had a tendency to produce more chemokines than β-glucan mice, an observation compatible with greater inflammatory cell recruitment and consistent with the findings from our peritonitis model. Interestingly, CXCL2 was the only chemokine that was produced more in β-glucan mouse mammary gland tissue (**Figure 8G**). No such observation was made during the peritoneal challenge. Histological examination of the mammary glands confirmed that PBS mice had a more intense inflammatory reaction and recapitulated the above findings. Indeed, PBS mouse mammary tissue was much more highly infiltrated by neutrophils than that of β-glucan mice (**Figure 8H**, insert 1), showing visible clusters of bacteria (insert 2) and necrotic intraductal alveolar cells (insert 3).

## Discussion

Overall, our preclinical study indicate that β-glucan supplementation allows better control of *E. coli* peritoneal infection, as shown by a lower number of CFUs in the liver and spleen and partial protection from necrosis-associated tissue damage. As stated in the introduction, β-glucan sources are numerous and highly influence their biological properties (34), including those of immune modulation. In the current study, we used β-1,3/1,6 branched glucans from the yeast *Saccharomyces cerevisiae* and for the sake of clarity, we will discuss our results in light of studies that used the same source, and not those that used β-glucans from cereals, bacteria, or higher fungi (mushrooms).

These previous studies showed that following a nonlethal primary infection with *Candida albicans*, a pathogenic yeast with a β-glucan-rich cell wall, mice were protected from a secondary, supposedly lethal, re-infection (35). More specifically, the transfer of plastic-adherent innate immune cells (macrophages) collected from mice primarily challenged with *C. albicans* into compatible recipient mice provided them considerable protection against a secondary challenge. It was subsequently confirmed that such protection was independent of lymphocyte activity (and memory) by using Rag knock-out mice, but was, in contrast, related to monocytes, as *Ccr2* knock-out mice were not protected (36). Further experiments demonstrated that the mechanism relied on β-glucan recognition by monocytes through their cognate receptor, dectin-1, and led to the hyper-production of inflammatory cytokines *in vitro* and *in vivo* during a secondary challenge (36). However, in our study, there was no evidence of increased cytokine production after peritoneal challenge with *E. coli* in mice that received oral β-glucans. Indeed, cytokine quantification at steady state and throughout the infection trial showed that, instead, β-glucan -supplemented mice produced even lower amounts of cytokines in peritoneal exudates than PBS control mice, especially at later time points. No differences were observed between the control and treated groups at early time points. However, at 8 h p.i., the β-glucan group showed high heterogeneity among individual responses, in which half of the mice had already downregulated their cytokine production, as shown in the clustering analysis. Although we cannot exclude that the peak of secretion occurred earlier, i.e. already between 4 and 8 h p.i., the observed BAFF secretion kinetics makes this hypothesis very unlikely. Indeed, in contrast to the other cytokines measured, BAFF accumulated throughout the infection, from basal to high secretion levels, especially in the PBS mice. In the β-glucan mice, BAFF secretion remained low but progressively increased for all mice, and no secretion peak could be found, even at the peak of neutrophil recruitment (8 h p.i.). It was recently demonstrated that neutrophils are a major source of BAFF, further exacerbating the inflammatory process (30), consistent with the pattern of response observed in the PBS mice. Overall, our data confirm that β-glucan supplementation by the oral route enables rapid mitigation of inflammatory cytokine production.

Consequently, the recruitment of neutrophils in peritoneal exudates initiated shortly after *E. coli* infection (before 8 h p.i.) was rapidly switched off relative to that of PBS mice, which showed exacerbated infiltration in both exudates and the peritoneum, as shown by the flow cytometry and histology results. This again demonstrates more rapid resolution of the inflammatory response in β-glucan-supplemented mice.

In parallel, the proportion of inflammatory monocytes increased in β-glucan mice, but this was more likely a consequence of the faster resolution of the inflammatory response and the decline in neutrophil numbers rather than a direct effect of β-glucans. Consistent with this hypothesis, the absolute numbers of inflammatory monocytes did not very differ between the two experimental groups. Nevertheless, flow cytometry demonstrated that monocytes infiltrating the peritoneal cavity differentiated into F4-80^high^ macrophages more rapidly in β-glucan than PBS mice. Again, this observation was likely related to the faster mitigation of the inflammatory response, leading to highly different cytokine contexts for the conditioning of monocyte differentiation and other properties. However, differences in the activation status or pro-resolving properties of these inflammatory monocytes cannot be completely excluded.

β-glucan-induced immune training is associated with both epigenetic and metabolic reprogramming of monocytes, leading to the over-production of inflammatory cytokines during a secondary challenge (i.e., LPS) (37). Moreover, it was previously demonstrated that mouse peritoneal macrophages (either resident or thioglycolate elicited) or BMDM (23) and bone-marrow monocytes (38) can be trained *in vitro* using β-glucans. To test this feature in our *in-vivo* model, we isolated F4-80^high^ resident peritoneal macrophages from β-glucan-supplemented or PBS control mice and further stimulated them *ex vivo* with LPS. No differences in either TNFα or IL-6 production were observed. The analysis of this population was consistent with the peritoneal infection route but it was perhaps not the best choice to evaluate monocyte training. Indeed, the F4-80^high^ resident macrophage compartment is replenished by circulating monocytes after infectious or inflammatory insults (26), but such turnover is unlikely to occur in a confined animal facility.

Recently, it was shown that β-glucan administration can protect against *Mycobacterium tuberculosis* infection by increasing the cytokine responses of monocytes and expanding hematopoietic stem and progenitor cells in the bone marrow and increasing myelopoiesis (39).This finding shows that β-glucans can modify the monocyte response already in the early stages of bone-marrow differentiation. To test this possibility this, we generated macrophages starting from bone-marrow precursors isolated from β-glucan- or PBS-supplemented mice using M-CSF and stimulated them with LPS. However, we observed no difference in cytokine secretion between both groups. Yet, it is possible that differentiation *via* M-CSF conditioning for six days may have altered the trained phenotype of the stem cells. Indeed, the effect of M-CSF and GM- CSF on the immune training of monocytes is still debated. Priming human peripheral monocytes in an M-CSF context, considered to be anti-inflammatory, upregulates β-glucan-mediated immune training and leads to strong cytokine secretion, whereas the opposite is found with GM-CSF (40). On the contrary, priming bone-marrow M-CSF-derived macrophages with GM-CSF before β-glucan exposure significantly enhances their training potential (38). To avoid any conditioning interactions with colony-stimulating factors, we sorted bone-marrow monocytes untouched by depletion and then directly stimulated them with LPS. Again, we observed no differences in the cytokine secretion between the two experimental groups. Although we did not check for epigenetic marks or changes in metabolism, these experiments argue that oral β-glucans supplementation did not induce monocyte training, at least with respect to inflammatory cytokine hyper-secretion, considered to be a hallmark in *in-vitro* differentiation models (41). Of note, the β-glucans used in the present study were demonstrated as efficient to induce murine macrophages training *in vitro* (23, 38). However, a recent study demonstrated yeast β-glucans reshaped IL-4 polarized macrophages into chemokines rather than inflammatory cytokines producers (42). Although chemokines were not determined in *ex vivo* re-stimulated macrophages supernatants, this finding is compatible with the precocious and transient neutrophils recruitment in β-glucans supplemented mice. Similarly, the adoptive transfer of macrophages trained *in vitro* with β-glucans in mice challenged with Pseudomonas aeruginosa improved bacterial clearance and promoted recruitment of inflammatory cells including neutrophils and monocytes (43). More investigations are needed to fully ascertain the contribution of macrophages in the protection granted *in vivo* following supplementation with β-glucans. Based on this study’s results and known features of macrophages trained *in vitro*, further experiments should be settled to finely evaluate phagocytosis and killing of bacteria after oral supplementation with β-glucans.

Comparison of the numbers of precursor for either neutrophils or monocytes in the bone marrow at steady state showed them to be similar between the two groups. This result is not in accordance with those of two recently published studies that highlighted the hematopoietic effect of β-glucans. However, the reported mechanism relied on the production of innate immune mediators, such as IL-1/IL-1b and GM-CSF, resulting from the administration of β-glucans by the i.p. route (39, 44). It has long been known that the intraperitoneal administration of zymosan, a particulate compound extracted from the yeast cell wall and containing β-1,3/1,6 glucans, induces peritonitis and, consequently, the release of inflammatory cytokines, including IL-1β (45). In our model, no signs of inflammation were present after the supplementation period, an observation that has been consistently reported in studies and clinical trials that administrated β-glucans by the oral route (reviewed in (46).

It is, therefore, important to compare studies that not only used yeast as the source of β-glucans but also used oral administration as the delivery route. Indeed, administration of β-glucans by the intravenous, intraperitoneal, or subcutaneous routes has been shown to increase the magnitude of the effects recorded, making it easier to decipher the underlying mechanisms involved. Moreover, β-glucans are recognized by numerous leukocytes through their interaction with dectin-1, which leads to cell activation. Consequently, systemic injections of such active compounds leave the organism in a state of reactiveness that could explain how protection is achieved. By contrast, the numerous clinical trials conducted in humans using dietary/oral β-glucans consistently report far more subtle effects, such as better resistance to seasonal infections or a reduction in allergies, but, strikingly, with very few or no evident changes in immune parameters, including plasma cytokine levels (reviewed in (14, 46)). Moreover, *ex-vivo* stimulation of peripheral blood mononuclear cells isolated from patients who received 1 g of oral β-glucan supplementation daily failed to show any increase in cytokine production (47). Overall, these studies raise questions concerning the bio-availability of β-glucans and their capacity to interact at the molecular or cellular level with the immune system when delivered by the oral route. Nevertheless, several studies find β-glucans in the plasma after oral delivery and show their translocation either through gastrointestinal cells and M cells from Peyer’s patches (19) or internalization by dendritic-cell projections through the gut epithelium (48). Importantly, such demonstrations of the passage of oral β-glucans were achieved using soluble β-glucan, whereas those used in the present study were insoluble and, thus, formed particles. Closer to our preclinical model, it has been shown in mice that particulate β-glucans from yeast are phagocytosed by macrophages and dendritic cells present in the M cell pocket and further disseminated to the lymph nodes, spleen, and even bone marrow (20, 21). Here, using a very similar β-glucan source, we did not find any evidence of a higher activation state or training by cells isolated from β-glucan supplemented mice, with or without differentiation, despite the possibility of orally delivered β-glucans interacting with bone marrow and priming stem cells or resident monocytes.

The discrepancy between the large number of studies reporting a positive effect of oral supplementation with yeast β-glucans against viral or bacterial infections and the scarcity of immunological evidence directly associated with it clearly demonstrates the absence of a known link between the reported effects and the mechanism of action for the general mechanism of immune training.

Although the mechanism is yet to be elucidated, our study strongly suggest that the protection granted by oral supplementation with β-glucans does not result from an increased inflammatory response but, rather, its better downregulation. Moreover, it appears that such protection is provided throughout the body, as elegantly shown by our complementary intra-mammary *E. coli* infection model. Indeed, following β-glucan supplementation, mice with either systemic peritoneal or local mammary infections showed highly similar reactions, including the dampening of bacterial fitness, a strong reduction in tissue damage, and the downregulation of inflammatory processes.

Interestingly, following intra-mammary *E coli* challenge, CXCL2 production was significantly higher in β-glucan mice, whereas PBS mice instead produced CXCL1. These two chemokines have different cellular sources and play distinct roles in promoting neutrophil recruitment and stimulating their antibacterial activities. Cell-depletion experiments have demonstrated that neutrophils significantly contribute to the *in-vivo* production of CXCL2 but not CXCL1. Neutrophil-derived CXCL2 acts in an autocrine manner to increase its own production and enhance antibacterial activity, including the release of oxygen reactive species (49). These elements may explain how better control of the bacterial burden was achieved in β-glucan-supplemented mice in our coliform infected mammary gland model. However, such a difference was not observed in the coliform peritonitis model, probably due to the high inter-individual variations in the β-glucan group, especially at 8 h p.i., at which time half of the mice had already downregulated their cytokine production. Of note, although dectin-1 is considered as central in β-glucans sensing by macrophages, others receptors including scavengers receptors, lactosylceramide, some Toll Receptors but also the Complement Receptor 3 (CR3) can bind β-glucans (Reviewed in (46)). While Dectin-1 is mainly expressed by macrophages and dendritic cells, CR3, in turn, is highly expressed by neutrophils. This is to be considered for future studies as macrophages may not be the only cell population to be trained or primed following interaction with β-glucans. Indeed, *in vitro* studies have established that neutrophils derived from Bacillus Calmette Guérin (BCG)-vaccinated individuals show a trained phenotype with increased expression of CR3 and IL-8 following re-stimulation with BCG unrelated stimuli (50) confirming that neutrophils can effectively be trained. Whether β-glucans can train neutrophils remains to be determined.

How β-glucans supplementation promotes resolution after E. coli infection is yet to be deciphered but our data strongly suggest that immune training of monocytes/macrophages is not necessarily involved and that other functions contribute to the observed protection against detrimental infections. This study is the first to show that oral β-glucans can considerably alleviate the severity of acute mammary gland infection induced by living *E. coli* (coliform mastitis). Consistent with our findings, oral supplementation with β-glucans reduced inflammatory cytokines, MPO, and iNOS levels following LPS-induced mastitis in a previous study in rats (51). More recently, β-glucan supplementation of dairy cows – in which mastitis is a major udder health problem - did not improve milk somatic cell counts, an indicator of mammary gland health, nor daily milk production or protein and fat content (52). However, as no immune parameters were measured and no experimental challenge performed in the later study, it is difficult to reach any definitive conclusions.

In conclusion, additional mechanistical studies, using clearly defined β-glucan sources, are needed to establish how oral supplementation grants protection against severe bacterial infections. This is a prerequisite before clinical translation of these exciting findings can be persued.

## Data availability statement

The raw data supporting the conclusions of this article will be made available by the authors, without undue reservation.

## Ethics statement

The animal study was reviewed and approved by Ethics Committee on Animal Experimentation National Veterinary School of Toulouse.

## Author contributions

SW: Conception, Investigation, Analysis, Original manuscript writing KB: Conception, Investigation, Analysis, Reviewing and editing final manuscript TS: Investigation, Analysis CC: Investigation, Analysis LG: Reviewing and editing final manuscript EM: Supervision, Reviewing and editing final manuscript GF: Supervision, Funding, Reviewing and editing final manuscript GT: Conception, Investigation, Analysis, Supervision, Funding, Original manuscript writing. All authors contributed to the article and approved the submitted version.
