# Supplementary material for: Oral supplementation with yeast β-glucans improves the resolution of Escherichia coli-associated inflammatory responses independently of monocyte/macrophage immune training
Source: Front Immunol. 2022 Dec 20;13:1086413. doi: 10.3389/fimmu.2022.1086413 (PMC9809295; doi:10.3389/fimmu.2022.1086413)
Supplement: Supplementary file 1 [file DataSheet_1.pdf]

A

B

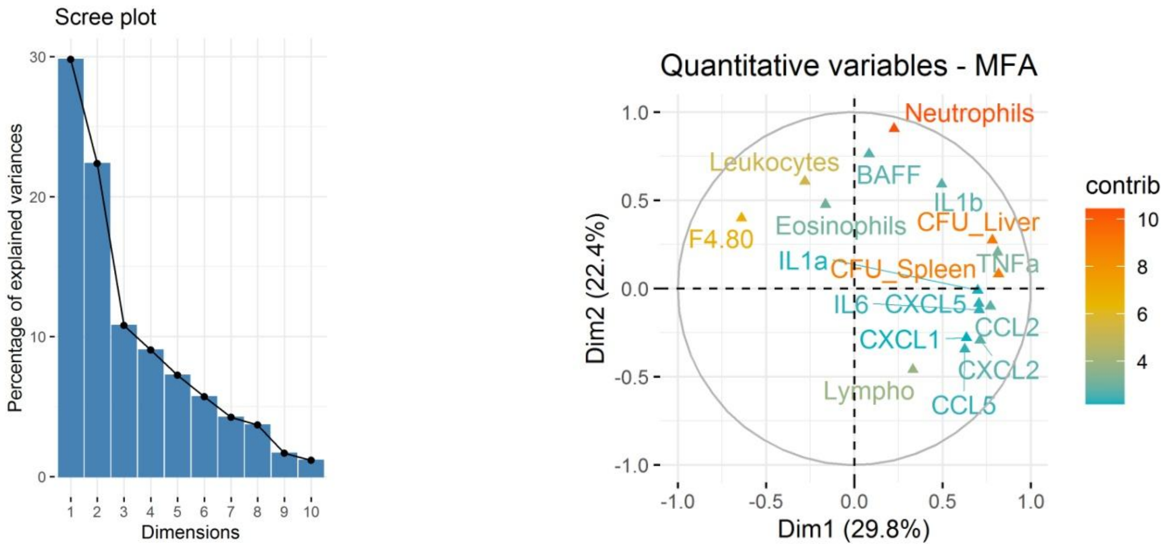

Supplemental Figure 1:

A – Scree plot displaying the percentage of explained variances by the various dimensions

B - Contribution of quantitative variables to dimensions Dim1 and Dim2

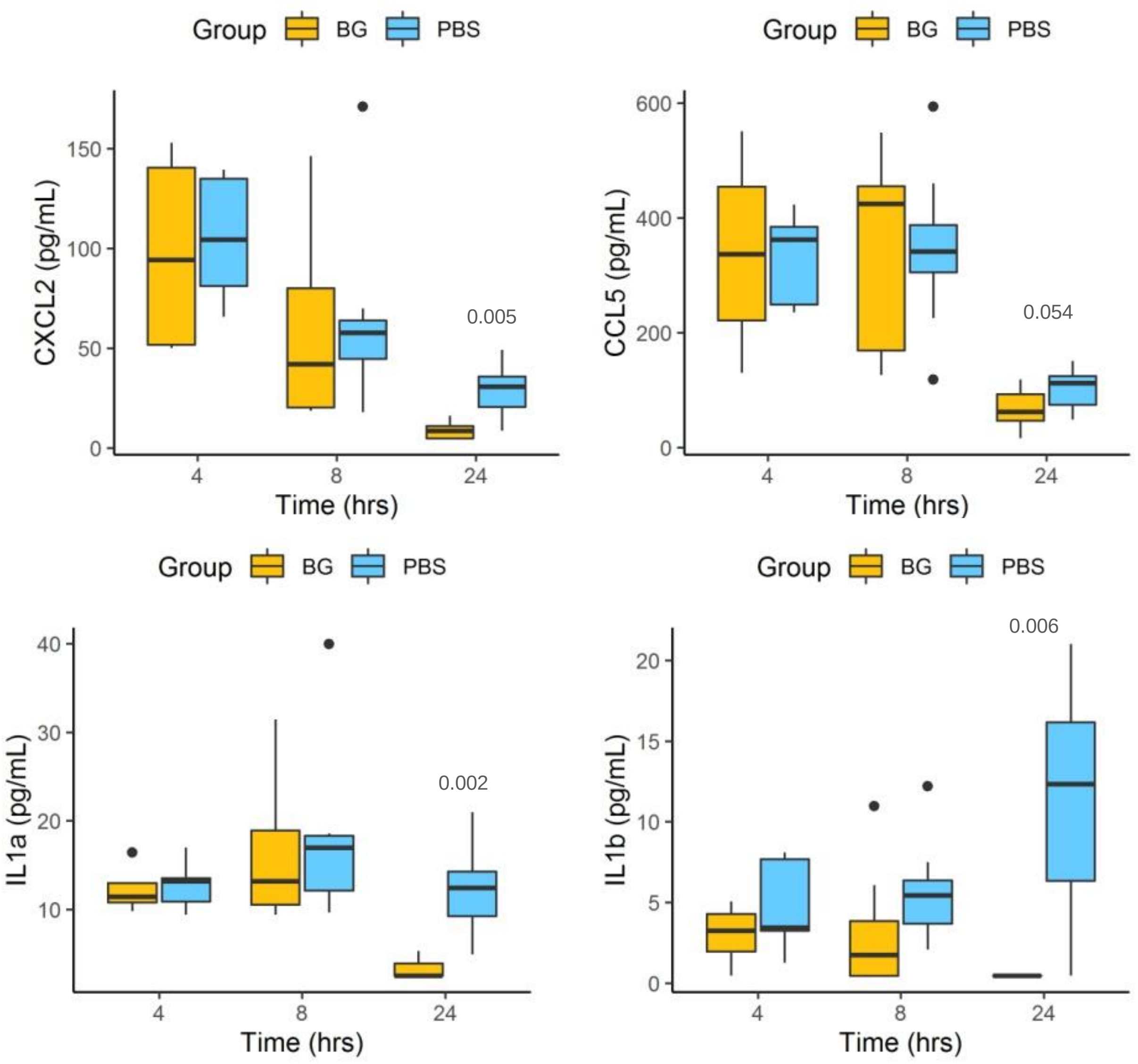

Supplemental Figure 2: Multiplex ELISA measurement of cytokines secretion in peritoneal exudates. Statistical analysis was performed using Welch t Ttest. When significant or near signification p values are indicated .

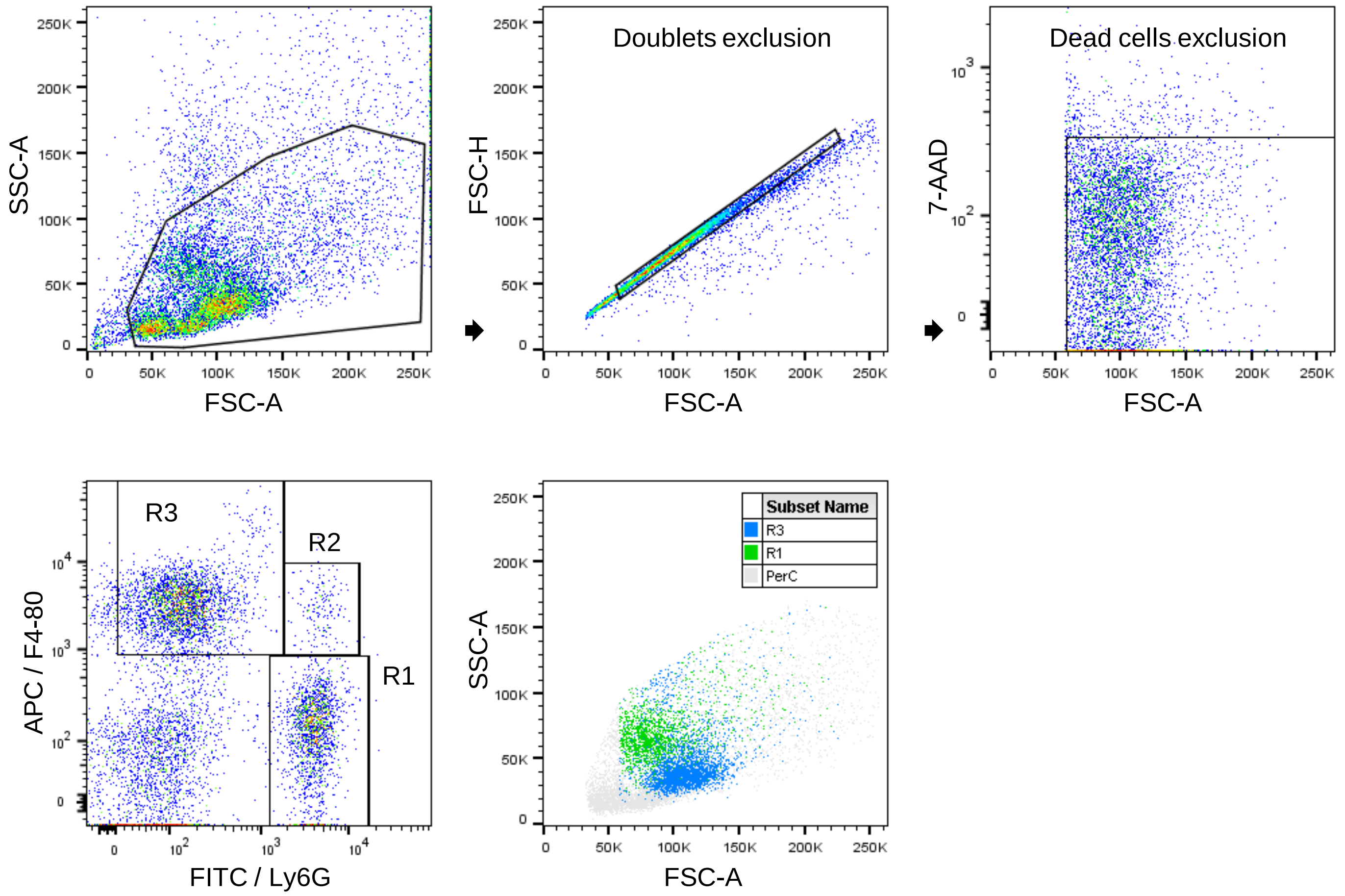

Supplemental Figure 3: Flow cytometry gating strategy for labeling and analysis of Neutrophils (R1) ; F4-80^{pos}(R3) ; Efferocytosis (R2) in peritoneal exudates. Polymorphonuclear neutrophils (PMNs) were considered as Ly6G^{pos} F4-80^{neg} cells present in R1. Cells from R3 were, at this step of the study ,considered as F4-80^{pos} as other markers were needed to establish a clear phenotype.

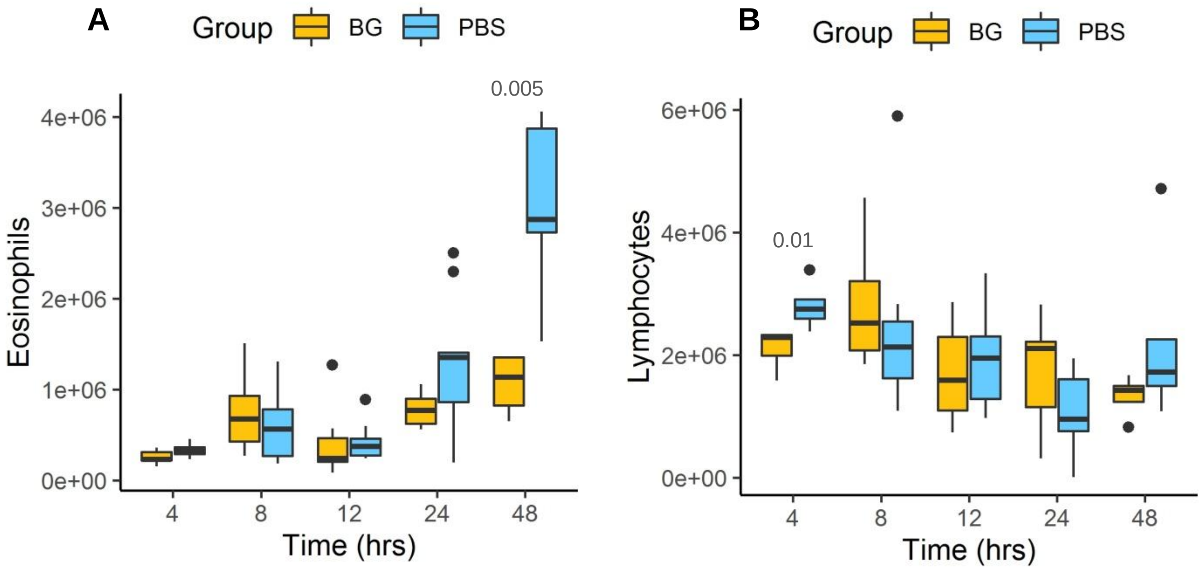

Supplemental Figure 4: Flow cytometrical analysis of eosinophils and lymphocytes dynamics in peritoneal exudates from BG and PBS mice. Statistical analysis was performed using the Welch t Test.

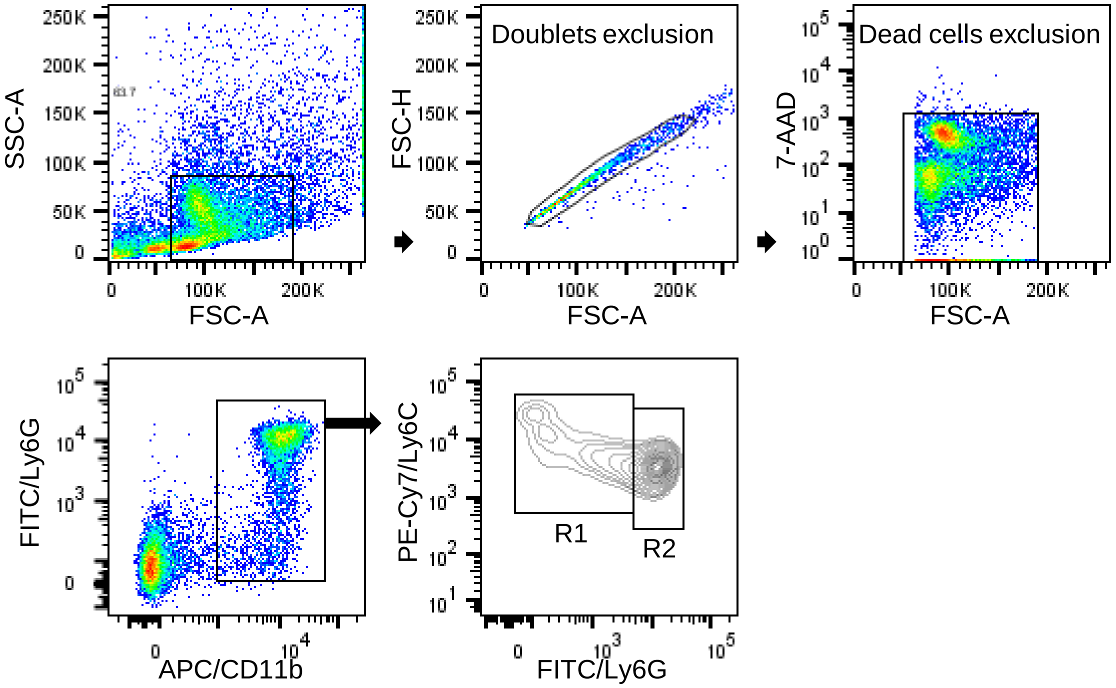

Supplementary Figure 5: Flow cytometry gating strategy for labeling and analysis of Bone marrow Neutrophils (R1): CD11b^{pos} Ly6C^{High} Ly6G^{Low}= immature; (R2): CD11b^{pos} Ly6C^{Low} Ly6G^{High} =mature

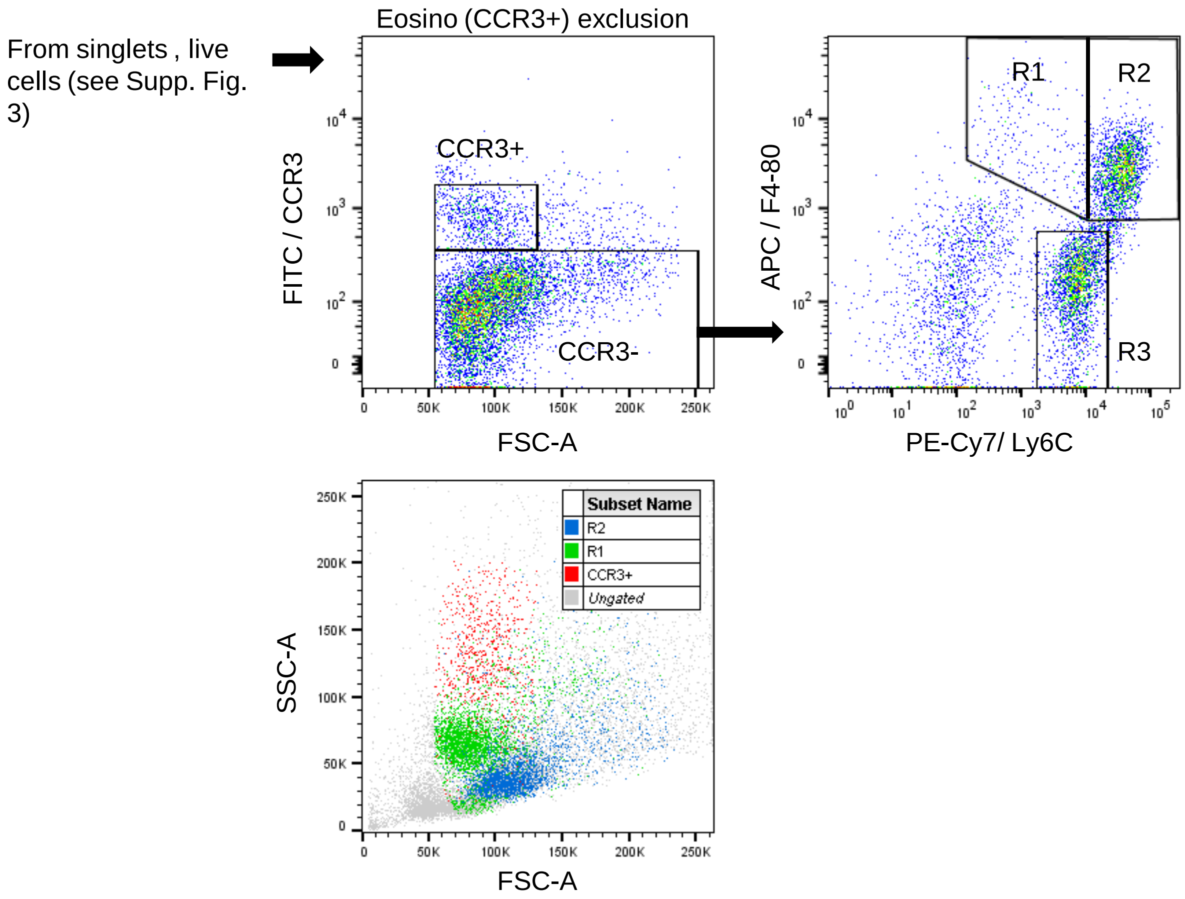

Supplemental Figure 6: Flow cytometry gating strategy for labeling and analysis of Monocytes CCR3^{neg}, Ly6C^{high}, F4-80^{low} (R2) ; Macrophages CCR3^{neg}, Ly6C^{low} F4-80^{high} (R1) ; CCR3^{neg}, Ly6C^{low} F4-80^{Neg} (R3) as neutrophils in peritoneal exudates. CCR3^{pos} SSC^{high} eosinophils were gated out. From total cells, we removed cell debris and dead cells and eliminated cell doublets. Finally, we excluded eosinophils (by using CCR3 marker) as some of them can share the F4-80 marker with macrophages populations. The remaining cell populations are then considered as 100% and include R1 + R2 + R3 gates + ungated cells.

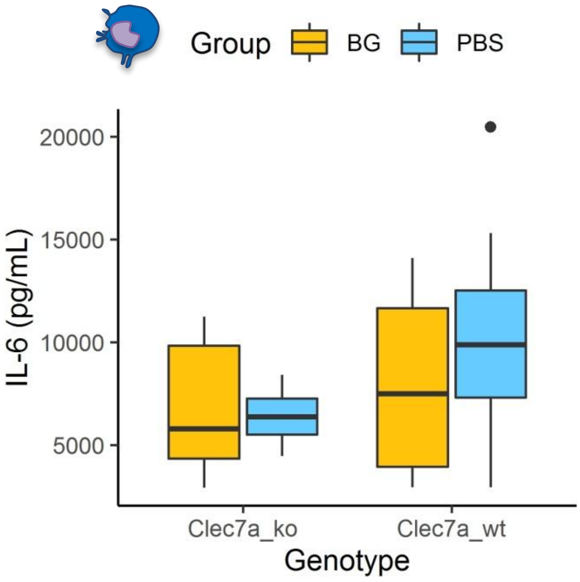

Supplementary Figure 7: IL-6 concentrations from bone marrow isolated monocytes stimulated with LPS determined by ELISA method.Statistical analysis was performed using Welch t Test.

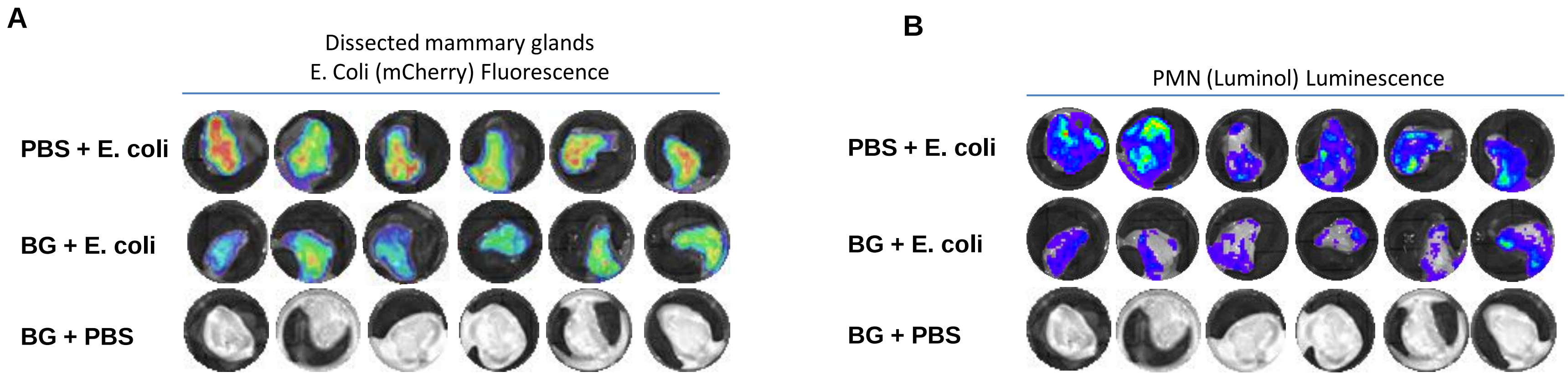

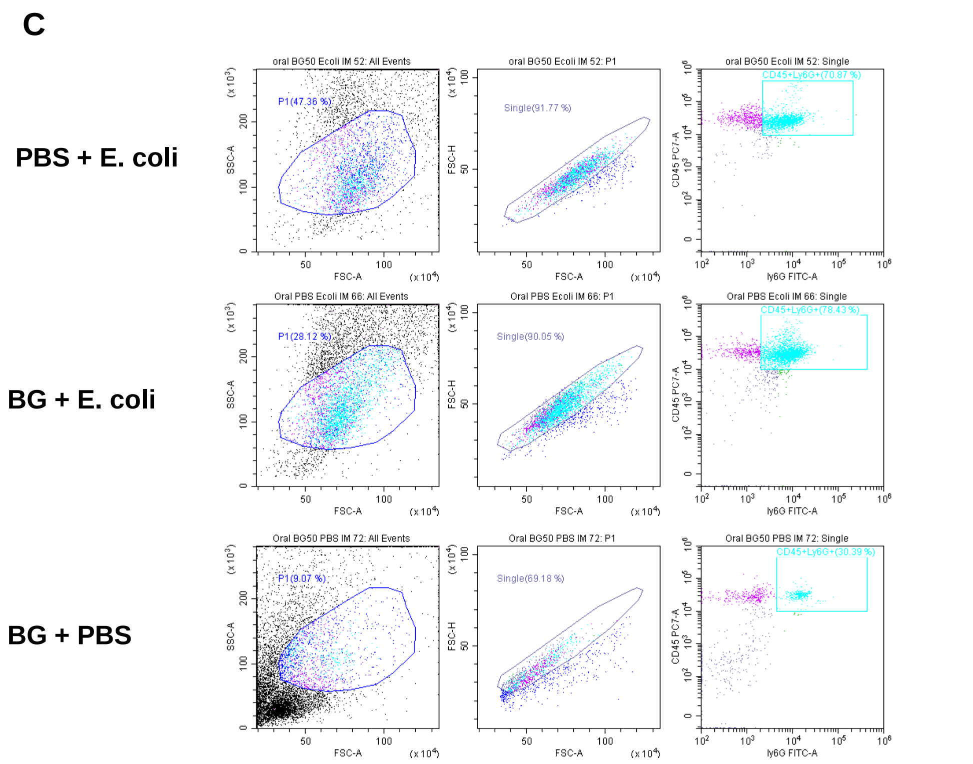

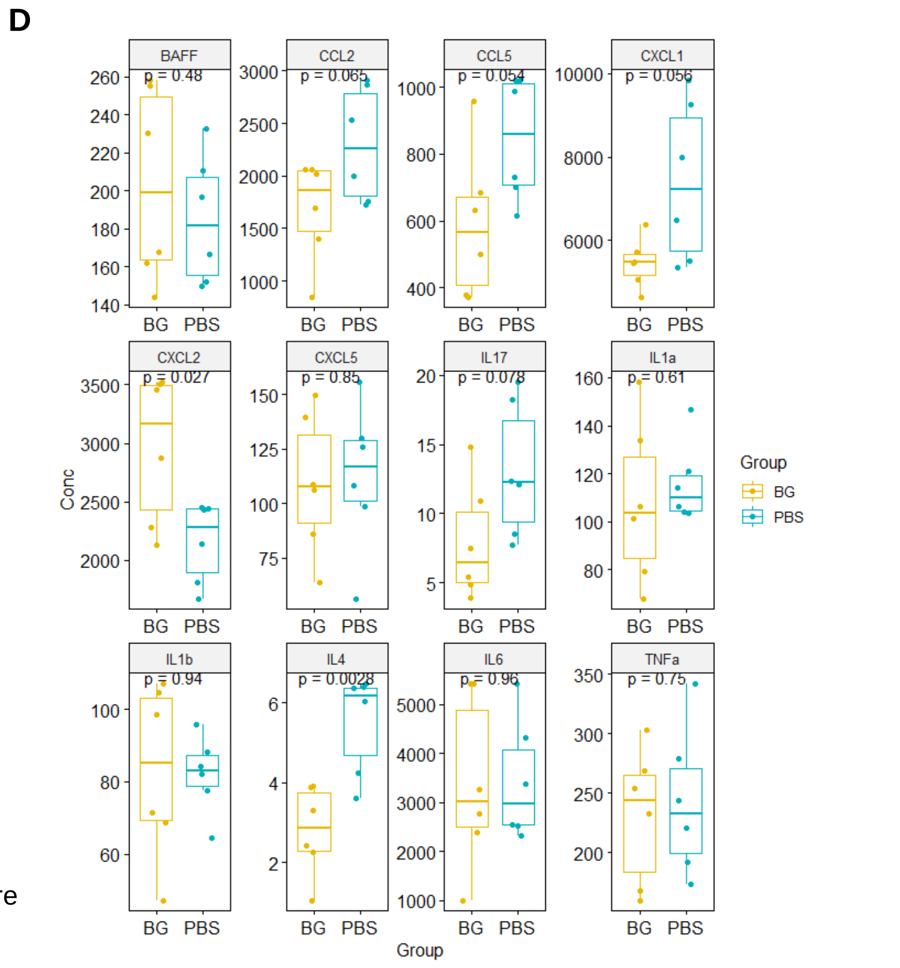

Supplementary figure 8: 6 mammary glands were dissected from 3 mice of each group and analyzed for fluorescence (A) and luminescence (B). Mammary glands were digested and cell suspensions analyzed in flow cytometry (C). Cytokines were measured in tissue lysates (D)

Group	Time	Mice per exp.	Individual exp.	Total mice
BG	4	5	1	5
	8	4	2	8
	12	5	2	10
	24	4/5	2	9
	48	5	1	5
PBS	4	5	1	5
	8	4	2	8
	12	5	2	10
	24	4/5	2	9
	48	5	1	5

Supplementary table 1:

Experimental setting indicating numbers of individual experiments and mice numbers used for every time point of E. coli intraperitoneal challenge kinetics
